# Administration of granulocyte-macrophage colony-stimulating factor enhanced chimeric antigen receptor T-cell expansion and cellular immunity recovery without inducing cytokine release syndrome

**DOI:** 10.3389/fmed.2022.1042501

**Published:** 2022-11-03

**Authors:** Ying Jiang, Dan Feng, Chun Wang, Yanlei Zhang, Chuxian Zhao, Su Li, Youwen Qin, Alex H. Chang, Jun Zhu

**Affiliations:** ^1^Department of Hematology, Shanghai Zhaxin Traditional Chinese and Western Medicine Hospital, Shanghai, China; ^2^Shanghai YaKe Biotechnology Ltd., Shanghai, China; ^3^Department of Laboratory, Shanghai Zhaxin Traditional Chinese and Western Medicine Hospital, Shanghai, China; ^4^Clinical Translational Research Center, Shanghai Pulmonary Hospital, Tongji University School of Medicine, Shanghai, China

**Keywords:** chimeric antigen receptor T-cell, granulocyte-macrophage colony-stimulating factor, neutropenia, cytokine release syndrome, infection

## Abstract

**Background:**

Neutropenia and cytokine release syndrome (CRS) are two major toxicities of chimeric antigen receptor (CAR)-T cell therapy. Granulocyte-macrophage colony-stimulating factor (GM-CSF) is an ideal candidate treatment for neutropenia except for its potential aggravation of CRS. We hypothesized that the optimal timing of supplemental with GM-CSF in a shortage of host immunity and CAR T-cell was chosen as avoidance of CRS. In the study we evaluated the safety and efficacy of GM-CSF intervention post-CAR T-cell therapy while circulating CAR T-cell declined.

**Materials and methods:**

Nine patients received GM-CSF therapy who displayed moderate neutropenia with absolute neutrophil counts (ANC) < 1,500 cells/mm^3^ with concomitant declination of circulating CAR T-cell.

**Results:**

The median duration of GM-CSF intervention was 15 days (4–30). CAR T-cell expansion was observed in peripheral blood (PB) of seven patients (7/9). The median baseline and peak CAR T cells count in PB of the seven patients with CAR T-cell expansion were 0.85 × 10^6^/L (0–50.9) and 6.06 × 10^6^/L (1.43–112.55). And the peaks of CAR T-cell levels in PB appeared in day 7 (2–11) following the initiation of GM-CSF administration with increases of 2.84 × 10^6^/L (0.38–61.65). Also, increased white blood cells in PB were observed in all patients. The median onset and duration time of WBC recovery were 9 (1–14) and 17 (3–53) days. Moreover, the increment of WBC, neutrophil, lymphocyte and CD3-CD16 + CD56 + natural killer cell in PB was observed. In addition, no CRS or fatal infection occurred during GM-CSF treatment.

**Conclusion:**

This study provides evidence for the clinical feasibility of combining CAR T-cell therapy with the GM-CSF to treat neutropenia patients with concomitant declination of circulating CAR T-cell.

## Introduction

Chimeric antigen receptor T-cell (CAR T) therapy has been shown to be a promising remedy in varieties of hematological cancers (1–4). However, the efficacy of CAR T-cell therapy is limited by lack of persistence of CAR T cells and side effects associated with CAR T-cell infusion, including neutropenia, infection and cytokine release syndrome (CRS). Therefore, the overall management of CAR T-cell persistence, neutropenia recovery, as well as CRS intervention is urgently needed to enhance the long-term remission, prevent the life-threatening infection, as well as to alleviate the CRS and neurotoxicity associated with CAR T-cell therapy (5).

Granulocyte-macrophage colony-stimulating factor (GM-CSF) has been widely used in clinics for the treatment of severe neutropenia for patients receiving chemotherapy. Many researches have revealed that GM-CSF is superior to G-CSF (Granulocyte colony-stimulating factor) in priming polymorphonuclear leukocytes/peripheral-blood mononuclear cells (PMNLs/PBMCs) to mediated fungicidal activity and more effects on superoxide release from neutrophils (6–8). And GM-CSF can act in a paracrine fashion to recruit circulating neutrophils, monocytes and lymphocytes to enhance their functions in host defense (9). Also in the previous clinical trial, we have demonstrated administration of GM-CSF is an effective strategy not only in immune modulation, but also in reduction of fungal infections due to neutropenia following hematopoietic stem cell transplantation (HSCT) conditioning (10). Therefore, GM-CSF is an important hematopoietic growth factor and immune modulator (9).

Granulocyte-macrophage colony-stimulating factor (GM-CSF) is not recommended in the setting of CAR T-cell therapy in NCCN guideline because CRS was deteriorated by IL-6, IL-1 and nitric oxide produced by recipient macrophages in murine model (11). Meantime, our previous findings have showed the incidence of grades 2 to 4 acute GVHD in GM-CSF was more than that in G-CSF groups (30.9 vs. 21.7%), although there was no significant difference between the groups (*P* = 0.473) (10). These results also have provided the clue for GM-CSF probably activating CAR T-cells *via* macrophages. Then the remaining question is how about the delayed use of GM-CSF for patients to avoid the CRS and GVHD. However, to our knowledge, no clinical study has been reported in the beneficial effects of GM-CSF administration in patient’s undergone CAR T-cell treatment while circulating CAR T-cell declined.

Here we explored GM-CSF as a therapeutic intervention aimed at alleviating neutropenia, promoting cellular immunity recovery, as well as enhancing CAR T-cell expansion in patients. In this cohort, nine patients’ undergone different CAR T-cell therapies have been treated with GM-CSF, to assess the safety and efficacy of GM-CSF intervention post-CAR T-cell therapy.

## Materials and methods

### Study design

This study was designed to examine the safety and efficacy of GM-CSF in treatment of neutropenia post-CAR T-cell therapy at Shanghai Zhaxin Traditional Chinese and Western Medicine Hospital. The protocol was approved by the Hospital ethics committee (No. 202105). The primary objective of this study was to assess the safety of GM-CSF administration and the secondary objective was to evaluate the efficacy of GM-CSF intervention post-CAR T-cell therapy in alleviating neutropenia and enhancing the persistence of CAR T-cell. After CAR T-cell infusion, clinical outcomes including overall survival (OS) and relapse were evaluated up to date as of August 22 2022.

### Patients and chimeric antigen receptor T-cells

Patient’s undergone CAR T-cell treatments, who suffered from neutropenia with concomitant declination of circulating CAR T-cell were enrolled. The patients have received CAR T-cell therapies targeting CD7 surface receptor for one patient with T cell tumor and CD19, CD20, and CD22 surface receptors for other eight patients with B cell tumor. The CAR T-cell manufacture and detection were performed as previously reported (2, 3).

### Granulocyte-macrophage colony-stimulating factor treatment

Granulocyte-macrophage colony-stimulating factor (GM-CSF) was subcutaneous administrated. The indication for initiation of GM-CSF therapy was patients who display moderate neutropenia with absolute neutrophil counts (ANC) < 1,500 cells/mm^3^. The indication for termination of GM-CSF therapy was when their ANC levels had reached to 3,000–5,000 cells/mm^3^. Nine patients were started on the lowest available dose at 100 ug daily according to the instruction of recommended medication dosage once-daily subcutaneous GM-CSF 3 to 10 μg/kg/day (molgramostim, Topleucon; Xiamen Amoytop Biotech, Xiamen, China). After 3 days, the dose of GM-CSF in two patients (#6 and 9) was escalated to 200 ug daily because no increment in WBC was observed.

### Laboratory test

White blood cell (WBC), neutrophil, monocyte and lymphocyte were counted by Sysmex XN-1000 automatic hematology analyzer. BD Multitest™ 6-Color TBNK with Dxflex (Beckman) was used to determine the absolute counts of lymphocyte subsets in peripheral whole blood for immunophenotyping, including T lymphocytes (CD3 +), B lymphocytes (CD19 +), Natural killer (NK) lymphocytes (CD3–CD16 + CD56 +), Helper/inducer T lymphocytes (CD3 + CD4 +), and Suppressor/cytotoxic T lymphocytes (CD3 + CD8 +). The cytokine levels of patients were quantified by AimPlex microsphere based assay (Quantobio Co., Ltd.).

### Criteria

The criteria for assessment of therapeutic effects for acute lymphoblastic leukemia and lymphoma were defined in accordance with NCCN guidelines version 4.2021 (Acute Lymphoblastic Leukemia) and Lugano 2014. The severities of cytokine releasing syndrome (CRS) and immune effector cell-associated neurotoxicity syndrome (ICANS) were also assessed by NCCN guidelines version 4.2021 (Management of CAR T-cell-Related Toxicities).

### Statistical analysis

Statistical analysis was performed with SPSS software and Data Analysis version 23.0. Difference of continuous variables between different weeks was analyzed by paired two-tailed Student’s *t* test (cytokines and cell count). The Kaplan-Meier approach was performed to estimate overall survival. Data is considered to be statistically significant when *P* < 0.05.

## Results

### Clinical description of patients

Nine patients were enrolled in cohort from July 2021 and November 2021 to receive GM-CSF therapy whose clinical characteristics are shown in Table 1. The median age 39 (20–59) years old. Four patients had ALL and NHL in first to three relapse, five patients had primary refractory disease. One patient (#4) had relapsed after prior allogeneic hematopoietic stem cell transplant (allo-HSCT). Three patients (#3, 7, and 8) had undergone autologous HSCT (auto-HSCT). Three patients (#3, 7, and 9) had previously been treated with CAR T-cell. The median time of previous treatments was 435 (136–1613) days. Autologous CAR T-cell was infused in eight patients, while donor-derived CAR T-cell was infused in 1 post-allo-HSCT patient (#4). The median dose for CAR T-cell infusion was 1.2 (0.63–2.79) × 10^6^ cells per kilogram of body weight.

**TABLE 1:** Characteristics of patients.

No.	Gender	Age	Diagnosis	Prior therapy (day)	CAR-T				GM-CSF		Fever (cause)	Follow-up
						
					Disease status	Condition	Kind	Dose (10^6^/Kg)	After CAR-T (day)	Duration (day)		
1	male	23	B-ALL (ph-)	136	PD (extramedullary invasion)	FC	hCD19	1.2	8	14	N	died, PD
2	female	46	DLBCL	242	PD	CBV	hCD20	0.96	20	30	Y (pneumonia)	alive, PD
3	male	24	Burkitt lymphoma	363	PD	radiotherapy (10 Gy)	hCD19/22	0.63	10	18	N	died, PD
4	male	39	T-LBL	1,064	PD	FC	donor hCD7	2.79	32	10	Y (GM-CSF)	died, CR
5	male	35	B-ALL (ph +)	510	PD (CNS involvement)	FC	hCD19	1.14	17	12	Y (urinary tract infection)	alive, CR
6	female	20	PMBL	417	PR	FC	hCD19/22	1.03	21	15	Y (GM-CSF)	alive, PR
7	male	50	DLBCL	1,613	PD	FC	hCD22	1.24	126	4	N	alive, PR
8	male	51	PCNSL	435	NR	FC	hCD19	2.49	35	15	N	alive, PR
9	female	59	B-ALL (ph-)	1,152	CR4 (MRD +)	FC	hCD22	1.31	18	21	N	died, CR

ALL, acute lymphoblastic leukemia; Ph, philadelphia chromosome; DLBCL, diffuse large B cell lymphoma; LBL, lymphoblastic lymphoma; PMBL, primary mediastinal B cell lymphoma; PCNSL, primary central nervous system lymphoma; MRD, minimal residual disease; CNS, central nervous system; PD, progressive disease; SD, stable disease; PR, partial remission; NR, Non-remission; CR, complete remission with negative MRD; CR4, Fourth complete remission; FC, fludarabine and cyclophosphamide; CBV, cyclophosphamide bendamustine and etoposide.

**FIGURE 1:**
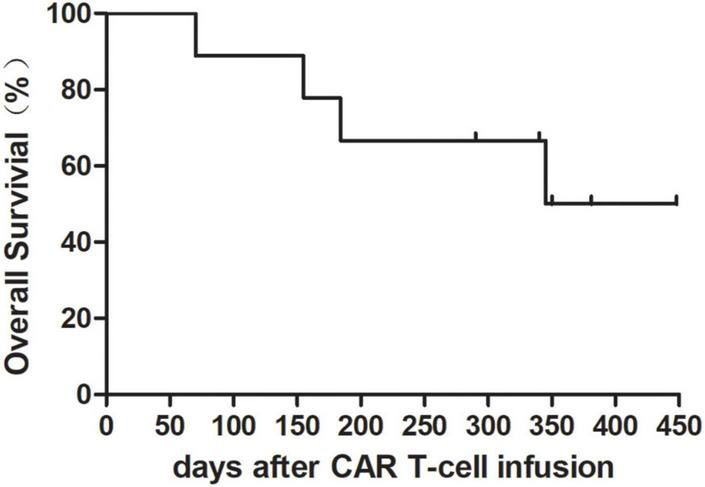
Overall survival rate after chimeric antigen receptor (CAR) T-cell infusion with granulocyte-macrophage colony-stimulating factor (GM-CSF). Overall survival rate in nine patients was 50% at 365 days following CAR T-cell infusion.

At a median observation time of 340 (70–448) days, two patients (#1 and #9) were bridged to allo-HSCT and patient No. 5 was bridged to auto-HSCT. Two patients (#1 and #4) died of infection at day 184 and 155. Cancer-related death occurred in patient #3 at day 70 following CAR T-cell infusion. Patient #9 died of graft-versus-host disease (GVHD) at day 345 after the date of CAR T-cell infusion. The clinical outcomes including overall survival (OS) and adverse effects were evaluated up to date of August 22 2022. The OS at 365 days following CAR T-cell infusion was 50% (Figure 1). Overall response rate (ORR) was 66.67% (6/9).

### Chimeric antigen receptor T-cell expansion post-granulocyte-macrophage colony-stimulating factor administration

**FIGURE 2:**
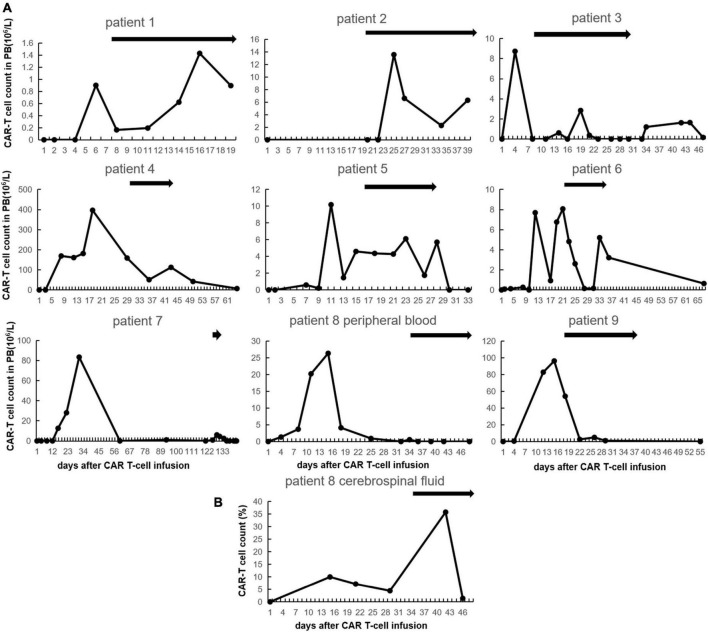
Chimeric antigen receptor (CAR) T-cell expansion after CAR T-cell infusion with granulocyte-macrophage colony-stimulating factor (GM-CSF). **(A)** CAR T-cell count in peripheral blood of each patient. The black bars indicate duration of GM-CSF using. **(B)** CAR T-cell count in cerebrospinal fluid of patient No. 8.

We serially evaluated CAR T-cell expansion in patients with GM-CSF. The median interval between start of GM-CSF and CAR T-cell infusion was 20 days (8–126) while the number of CAR T-cell in peripheral blood (PB) dropped to 20.3% (0–59.7) of pre-GM-CSF peak except for patient #2 who had no CAR T-cell expansion before GM-CSF administration (Figure 2A). The median duration of GM-CSF intervention was 15 days (4–30). Seven out of nine patients (77.8%) experienced CAR T-cell expansion in PB during GM-CSF usage. The median baseline and peak CAR T cells count in PB of the seven patients with CAR T-cell expansion were 0.85 × 10^6^/L (0–50.9) and 6.06 × 10^6^/L (1.43–112.55). The peaks of CAR T-cell levels appeared in day 7 (2–11) following the initiation of GM-CSF administration with increases of 2.84 × 10^6^/L (0.38–61.65). In addition, although no increased CAR T-cell count in PB was observed in patients #8 and #9, the percentage of CAR T-cell in cerebrospinal fluid of patient #8 increased from 4.43 to 35.78% at day 6 and dropped to 1.38% at day 10 meanwhile GM-CSF was administrated (Figure 2B).

### The reduction of partially inflammatory response

**FIGURE 3:**
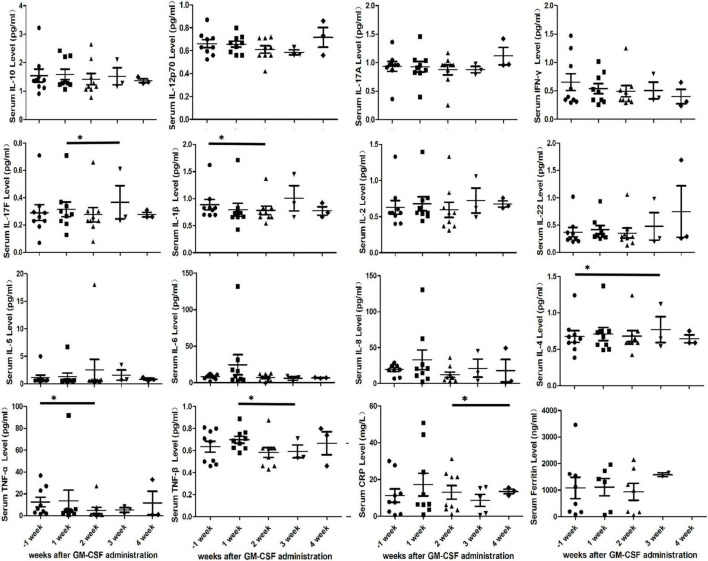
Cytokine profiles, Serum C-reactive protein (CRP) and ferritin in peripheral blood of nine patients after initial granulocyte-macrophage colony-stimulating factor (GM-CSF) administration. Serum cytokine profiles include IL-10, IL-12p70, IL-17A, IFN-γ, IL-17F, IL-1β, IL-2, IL-22, IL-4, IL-5, IL-6, IL-8, TNF-α, and TNF-β. Asterisks indicates *p* < 0.05.

Inflammatory markers, such as cytokines, C-reactive protein (CRP) and ferritin, were monitored in this study. However, no significant increment of inflammatory markers was observed. On the contrary, the statistically significant decreases in the mean of cytokines (IL-17F, IL-1β, IL-4, TNF-α, and TNF-β) and C-reactive protein (CRP) were seen at the 4-weeks follow-up (*P* < 0.05) (Figure 3). Furthermore, we also found the serum GM-CSF level in PB of patient No. 7 which remained within the normal range at the 2-weeks follow-up (7.7, 11, and 11.8, normal range 0–39 pg/ml).

### The increment of different kinds of white blood cells

We found increased white blood cells in PB of all patients with GM-CSF. The median time to achieve a WBC level above 3,000 cells/mm^3^ was 9 (1–14) days following the initiation of GM-CSF administration. Then there was only one patient (#2) whose WBC count never dropped below 3,000 cells/mm^3^ again. In the other eight patient’s (88.89%), the median duration of WBC recovery was 17 (3–53) days and then WBC count dropped below 3,000 cells/mm^3^.

**FIGURE 4:**
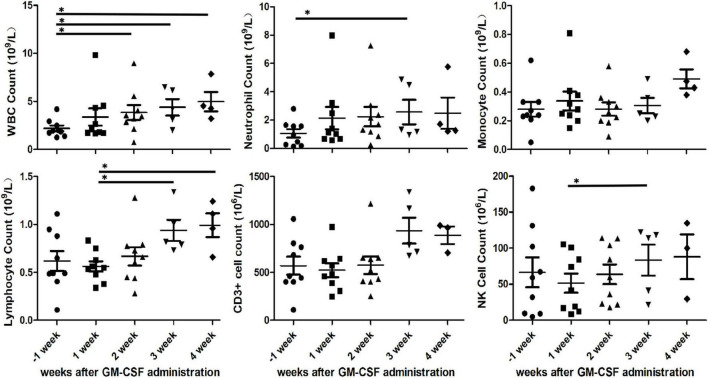
Various cells count in peripheral blood after initial granulocyte-macrophage colony-stimulating factor (GM-CSF) administration. White blood cell (WBC), neutrophil, monocyte, lymphocyte, CD3 + cell and CD3-CD16 + CD56 + natural killer cell in peripheral blood of nine patients. Asterisks indicates *p* < 0.05.

White blood cell count in PB of all patients gradually increased every week after GM-CSF administration. White blood cell count in PB prior to GM-CSF administration was 2.194 × 10^9^/L improved to 3.839 × 10^9^/L at the second week (*P* = 0.032, paired *t*-test), to 4.398 × 10^9^/L at the third week (*P* = 0.024, paired *t*-test) and to 4.965 × 10^9^/L at the fourth week (*P* = 0.025, paired *t*-test). Also, the increment of neutrophil, lymphocyte and CD3-CD16 + CD56 + natural killer cell was observed in PB. The mean of neutrophil count in PB every week before and after GM-CSF initiated were 1.066 × 10^9^/L, 2.134 × 10^9^/L, 2.239 × 10^9^/L, 2.564 × 10^9^/L, and 2.488 × 10^9^/L. There was statistically significant difference in neutrophil count between baseline and the third week (*P* = 0.037, paired *t*-test). Moreover, lymphocyte in PB was significantly increased at the third (*P* = 0.025, paired *t*-test) and fourth (*P* = 0.024, paired *t*-test) weeks compared to at the first week after GM-CSF administration. In addition, paired *t*-test statistical analysis revealed a significant increase in CD3-CD16 + CD56 + natural killer cell count between the first and third weeks (56.09 × 10^6^/L vs. 83.41 × 10^6^/L, *P* = 0.006, paired *t*-test). However, there were no significant difference observed in monocyte and CD3 + cell count at the baseline and 4-weeks follow-up (Figure 4).

### Adverse reactions during granulocyte-macrophage colony-stimulating factor administration

There were four patients who experienced fever while GM-CSF was administrated, of which two were GM-CSF related side effects, one was pneumonia and one was urinary tract infection.

From the first day of GM-CSF administration, patient #4 and #6 developed GM-CSF related fever without elevated cytokines and infection. Patient #4 experienced intermittent fever with highest temperatures up to 38.3°C during GM-CSF treatment and patient #6 have a high temperature up to 38.4°C during the first two days of the onset of GM-CSF administration. The patients’ body temperature was later dropped to normal range after physical cooling or loxoprofen sodium treatment. No cytokine release syndrome (CRS) or organ dysfunction or Immune Effector Cell-associated Neurotoxicity Syndrome (ICAN) was observed.

Infection occurred in two patients (2/9, 22.22%). One patient in this cohort (#2) had baseline neutropenia for 36 days and developed pneumonia for 6 days prior to GM-CSF administration (Table 1). The severity of the pneumonia in patient No. 2 was deteriorated from day 18 post-GM-CSF administration and the signs and symptoms of infection were alleviated 2 months later. In addition, another patient (#5) experienced mild urinary tract infection from day 5 post-GM-CSF administration.

## Discussion

It has been reported that there is 78% of patients developing Grade 3 or higher neutropenia in the ZUMA-1 trial (12) and 27% in the ZUMA-3 trial (13). It is recommended in NCCN guidelines that empiric broad-spectrum antibiotics, consider granulocyte colony-stimulating factor (G-CSF) if neutropenic after CAR T therapy. Granulocyte colony-stimulating factor (G-CSF) and granulocyte-macrophage colony-stimulating factor (GM-CSF) are both hematopoietic CSFs, the administration of them can reduce the duration and severity of neutropenia for patients. G-CSF is a relatively specific stimulator of the growth and differentiation of hematopoietic progenitor cells committed to the neutrophil lineage. However, the activity of GM-CSF in stimulating production and activation is not restricted to neutrophils because it also affects monocytes and eosinophils (10). Also, the application of GM-CSF is an immune adjuvant for its ability to increase dendritic cell (DC) maturation and function as well as macrophage activity. Although T cells do not possess a functional receptor for GM-CSF and thus do not rely on GM-CSF for proliferation and anti-tumor activity (14), one study in mice did show effect of GM-CSF on the anti-tumor properties of mouse CAR T-cell (15). In another study cancer cells transduced with an adenoviral vector carrying GM-CSF have induced strong antitumor immunity against tumor cells and prevented tumor regrowth in animal models (16). Therefore, we try to use GM-CSF to treat neutropenia in order to gain better anti-tumor effect of CAR T-cell and better host immunity against infection. In this study, therapeutic efficiency in combating neutropenia and increasing the number of CART cells without the occurrence of CRS were observed. Also, immune recovery was improved with GM-CSF administration.

Granulocyte-macrophage colony-stimulating factor (GM-CSF) is also known to increase the proliferation and activation of macrophages and blood monocytes, increasing their pro-inflammatory properties during infection (17). However, we find no significant difference observed in monocyte and CD3 + cell count at the baseline and 4-weeks follow-up. Our results showed white blood cell count in PB of nine patients gradually increased every week after GM-CSF administration. Also, the increment of neutrophil, lymphocyte and CD3-CD16 + CD56 + natural killer cell in PB was observed. The recovery of these cellular immunity improved anti-infective effects. Therefore, patient #5 whose infection occurred during GM-CSF using experienced mild symptoms and healed quickly. And patient #2 with baseline neutropenia 36 days before pneumonia diagnosis survived the life-threatening infection with administration of GM-CSF and augmented anti-infective therapy.

Of all the cytokines analyzed in the ZUMA-2 study, only peak levels of granzyme B and GM-CSF were associated with severe CRS and ICANS (18). Then the major question that remains to be answered is how to avoid CRS during GM-CSF therapy. Sentman et al. reported specific CAR T-cell effector mechanisms and the host immune system are required for this cytokine release-like syndrome in murine models. Cytokine release syndrome required two key effector molecules in CAR T-cell: perforin and GM-CSF (15). Moreover, Singh et al. demonstrated that GM-CSF is an exponential increase which secreted by monocyte-lineage cells upon CAR T-cell engagement of target tumor cells *in vitro* and in xenograft models (19). Therefore, the optimal timing of supplemental with GM-CSF in a shortage of host immunity and CAR T-cell was chosen as avoidance of CRS. And in this study we verified that GM-CSF implemented at the right time will be required to boost the expansion of CAR T-cell without inducing CRS.

Upon tumor engagement, CAR T-cell secrete pro-inflammatory cytokines such as GM-CSF and IL-8, which promote the release of the major CRS biomarker IL-6 as well as IL-8, cytokines that were previously shown to be monocyte-dependent (19). CRS biomarker release did not depend on physical contact between CAR T-cell and monocytes, suggesting that it may be possible to avoid CRS by lacking of sufficiently soluble factors involved in macrophage activation (14). In this study, no significant increment of inflammatory markers was observed during GM-CSF administration. Moreover, the statistically significant decreases in the mean of cytokines (IL-17F, IL-1β, IL-4, TNF-α, and TNF-β) and C-reactive protein (CRP) were seen during GM-CSF using. Data indicate that although GM-CSF levels are low in normal healthy individuals, activated lymphocytes produce elevated GM-CSF levels under pathologic conditions (20). But our results demonstrated increased lymphocytes with the serum GM-CSF level in PB within the normal range during GM-CSF administration. Thus, no CRS or ICAN in this study was observed because sufficiently soluble factors were unavailable.

Treatment with CAR T-cell resulted in an increased ability of local macrophages to kill tumor cells, which enhanced the anti-tumor effects of CAR T-cell therapy at the tumor site (21). GM-CSF increases macrophages’ responsiveness to CSF-1 (macrophage CSF) further enhancing the proliferation of resident macrophages in tissues (20). Other study reported GM-CSF activates resident microglial cells within the CNS, promotes blood–brain barrier (BBB) breakdown, and enables inflammation by other immune cells (22). Also, Sterner et al. reported that GM-CSF neutralization after CART19 reduced neuro-inflammation by 75% compared to CART19 plus isotype controls using human ALL blasts and human CART19 in this patient-derived xenograft mice model (5). However, it is not clear that this result can apply to human. Actually, in our study CAR T expansion in the cerebrospinal fluid, not in peripheral blood, was observed without CRS during GM-CSF therapy (patient #8). This result may be interfered with the baseline 10 Gy Whole-brain irradiation (WBI) 11 days prior to GM-CSF therapy and a second CAR T-cell infusion 2 days before GM-CSF administration.

## Conclusion

Overall, we explored the timing of GM-CSF administration to play a role in CAR T-cell expansion and cellular immunity recovery without inducing CRS which facilitates to increase durable complete responses and decrease infection rates after CAR T therapy. On the basis of these results, this approach may improve the overall safety and efficacy of CAR T therapies for cancer patients and may eliminate the need for robust anti-infection treatment. Therefore, this study provides evidence for the clinical feasibility of combining CAR T-cell therapy with the GM-CSF to treat neutropenia patients with concomitant declination of circulating CAR T-cell. However, these results are limited because of small population. Therefore, further research is needed to confirm these findings in this study.

## Data availability statement

The original contributions presented in this study are included in the article/supplementary material, further inquiries can be directed to the corresponding authors.

## Ethics statement

This study involving human participants were reviewed and approved by the Hospital Ethics Committee of Shanghai Zhaxin Traditional Chinese and Western Medicine Hospital (No. 202105). The patients/participants provided their written informed consent to participate in this study.

## Author contributions

YJ undertook the study design, treatment of patients, data analysis, and wrote this manuscript. DF performed the material collection. CW helped to interpret the data and revised the manuscript. YZ helped in technical support and manufacture of CAR-T cells. CZ and SL participated in treatment of patients. YQ performed the laboratory examination. AC helped in technical support and manufacture of CAR-T cells and revised the manuscript. JZ undertook project design and data analysis. All authors read and approved the final manuscript.
